# The ontogeny of social networks in wild great tits (*Parus major*)

**DOI:** 10.1093/beheco/arae011

**Published:** 2024-02-29

**Authors:** Sonja Wild, Gustavo Alarcón-Nieto, Lucy M Aplin

**Affiliations:** Cognitive and Cultural Ecology Research Group, Max Planck Institute of Animal Behavior, Am Obstberg 1, 78315 Radolfzell, Germany; Centre for the Advanced Study of Collective Behaviour, University of Konstanz, Universitätsstrasse 10, 78464 Konstanz, Germany; Department of Environmental Science & Policy, University of California Davis, One Shields Ave, Davis, CA-95616, USA; Cognitive and Cultural Ecology Research Group, Max Planck Institute of Animal Behavior, Am Obstberg 1, 78315 Radolfzell, Germany; Department of Migration, Max Planck Institute of Animal Behavior, Am Obstberg 1, 78315 Radolfzell, Germany; International Max Planck Research School for Quantitative Behaviour, Ecology and Evolution, Am Obstberg 1, 78315 Radolfzell, Germany; Department of Biology, University of Konstanz, Universitätsstrasse 10, 78464 Konstanz, Germany; Cognitive and Cultural Ecology Research Group, Max Planck Institute of Animal Behavior, Am Obstberg 1, 78315 Radolfzell, Germany; Department of Evolutionary Biology and Environmental Studies, University of Zurich, Winterthurerstrasse 190, 8057 Zurich, Switzerland; Division of Ecology and Evolution, Research School of Biology, Australian National University, 46 Sullivan’s Creek Road, Canberra, ACT 2600, Australia

**Keywords:** familiarity, great tit, ontogeny, *Parus major*, social networks, social stability, transition to independence

## Abstract

Sociality impacts many biological processes and can be tightly linked to an individual’s fitness. To maximize the advantages of group living, many social animals prefer to associate with individuals that provide the most benefits, such as kin, familiar individuals, or those of similar phenotypes. Such social strategies are not necessarily stable over time but can vary with changing selection pressures. In particular, young individuals transitioning to independence should continuously adjust their social behavior in light of developmental changes. However, social strategies exhibited during adolescence in animals are understudied, and the factors underlying social network formation during ontogeny remain elusive. Here, we tracked associations of wild great tits (*Parus major*) during the transition to independence and across their first year of life. Both spatial and social factors predicted dyadic associations. During the transition to independence in spring, fledglings initially preferred to associate with siblings and peers over non-parent adults. We found no evidence for preferred associations among juveniles of similar age or fledge weight during that time but weak evidence for some potential inheritance of the parental social network. By autumn, after juveniles had reached full independence, they exhibited social strategies similar to those of adults by establishing stable social ties based on familiarity that persisted through winter into the next spring. Overall, this research demonstrates dynamic changes in social networks during ontogeny in a species with a fast life history and limited parental care, which likely reflect changes in selective pressures. It further highlights the importance of long-term social bonds based on familiarity in this species.

## Teaser

Social networks of juvenile birds change dramatically during the transition to independence. While they initially prefer to associate with other juveniles, by autumn, their social strategies resemble those of adults. Like adults, first-year birds establish long-term social bonds with familiar individuals that persist over time, which highlights the importance of stable social bonds with others.

## INTRODUCTION

The quantity and quality of social bonds can directly affect various aspects of an individual’s fitness, including survival (Silk et al. 2010; Stanton and Mann 2012), reproduction (Cameron et al. 2009; Schülke et al. 2010), access to social information (Aplin et al. 2012; Atton et al. 2014), competition (Schoepf and Schradin 2012) and spread of diseases and parasites (Hamede et al. 2009; Bull et al. 2012). To maximize the advantages of group living, many social animals therefore, exhibit social strategies, interacting or associating more strongly with group members that provide the most benefits. These social strategies can follow different underlying rules, including preferences for kin (e.g., Gerlach et al. 2007; Wey and Blumstein 2010; Carter, Seddon, et al. 2013; Diaz-Aguirre et al. 2018), familiar individuals (e.g., Griffiths and Magurran 1999; King et al. 2011; Kohn et al. 2015; Keller et al. 2017), or those that share similar behavioral or phenotypical traits (e.g., Massen and Koski 2014; Carter et al. 2015; Bizzozzero et al. 2019; Langley et al. 2020).

Preferences for specific associates are not necessarily stable over time but can undergo temporal changes as a response to changes in external factors (e.g., Darden et al. 2009; Kelley et al. 2011; Leu et al. 2016), as well as an individual’s internal state (e.g., Chiyo et al. 2011; Blonder et al. 2012). For example, age-related changes in selective pressures can cause shifts in the benefits individuals receive from associating or interacting with specific individuals (Almeling et al. 2016; Murphy et al. 2020; de Lima and Ferreira 2021; Puehringer-Sturmayr et al. 2021). This effect has been well studied in primates, with individuals tending to shrink their social networks with age (Rosati et al. 2020; Siracusa et al. 2022). Yet, age-related changes also occur more widely. In wild cockatoos, for example, aging birds exhibit increased stability in social networks and increased longevity of bonds (Aplin et al. 2021).

Across all age groups, young individuals transitioning to independence are faced with perhaps the most drastically changing selection pressures at different stages of development and should, therefore, need to continuously adjust their social behavior to benefit from the advantages of group living (Silk 2007). For instance, in species with parental care, young individuals initially show strong associations with parental individuals, before establishing their own social network upon reaching independence (Gerber et al. 2019; Murphy et al. 2020; Muller et al. 2022). When reaching independence, young animals may then exhibit an increase in sociability, as for example as demonstrated in giraffes (Carter, Brand, et al. 2013). In mammalian species with maternal care, young individuals often inherit their mothers’ social network after independence, perhaps as they are more likely to establish social bonds with maternal contacts (Ilany and Akçay 2016). This has, for example, been demonstrated in African elephants (*Loxodonta africana*), in which daughters were found to inherit the social positions of their mothers (Goldenberg et al. 2016). In species in which bonds between parents and offspring are less central, however, the social strategies of young individuals—and in particular, how these change across development—remain more elusive. This holds true for songbirds: while often used as model systems for studying aspects of sociality, previous studies have largely focused on adults (e.g., Snijders et al. 2014; Welklin et al. 2023); but sociality during ontogeny has been largely ignored (but see Templeton et al. 2012; Franks et al. 2020).

Here, we describe the ontogeny of social networks in a songbird species during their first year of life in a species with limited periods of parental care, the European great tit (*Parus major*). Great tits are seasonal tree cavity breeders. In spring, the female will lay on average 6.5 eggs, and the breeding pair will raise their offspring in the nest for a period of approximately 22 days (Perrins 1979), after which chicks fledge from their nest (at which point they are referred to as “fledglings”). Fledglings then spend between 10–32 days in their family group, during which they continue to be fed by both parents (Verhulst and Hut 1996). After onset of independent feeding (at which point they are referred to as juveniles until their first possible breeding attempt at one year of age), they either integrate into local flocks or disperse (Dingemanse et al. 2003). While various studies have identified factors influencing social network position in both adult and first-year great tits—including body size (Farine et al. 2012) and personality (Aplin et al. 2013; Snijders et al. 2014; Johnson et al. 2017) — information on how young birds initially establish their social relationships and how social strategies change during ontogeny is lacking.

Here, we used established automated tracking techniques to follow the social associations of juvenile birds with other birds across their first year of life. First, we tracked birds from the point of fledging in late spring into summer, when juveniles leave their family groups and integrate into local flocks. We 1) quantify how the relationships with other birds (parents, non-parent adults, siblings, and non-sibling juveniles [peers hereafter] change during the transition to independence. We then investigate the more detailed factors underlying the establishment of social bonds. Natural selection pressures are particularly strong during the first few weeks after fledging due to increased predation risk and the struggle to find food after parents stop provisioning (Perrins 1979; Naef-Daenzer et al. 2001). This is particularly pronounced in lighter fledglings due to a lack of fat reserves. Juveniles should, therefore, exhibit social strategies that maximize access to the most relevant social information, and avoid competition. We therefore 2) test for potential homophilic preferences among juveniles, hypothesizing that individuals more similar in age and fledge weight may hold social information that is most relevant given developmental needs. Alternatively, we 3) investigate a potential inheritance of the parental social network, hypothesizing that familiarity may reduce competition.

Second, to investigate how social networks develop during the first year of life, we measured social associations for three-week periods during the subsequent autumn, winter, and spring. The winter months until the onset of spring are particularly challenging for first-year birds to find food, with mortality rates estimated at 43% versus 25% in old birds (Kluijver 1951). Since great tits rely extensively on social information for locating food sources (Aplin et al. 2012; Farine et al. 2015; Jones et al. 2017), first-year birds should benefit from associating more strongly with older birds who have already survived at least one winter. Conversely, the transition from winter to spring also coincides with the establishment of mating pairs, in which great tits tend to assort by age (Woodman et al. 2023). To better understand how these different selection pressures may drive social behavior, we therefore 4) tested for assortativity by age across seasons. Finally, great tits exhibit high breeding pair fidelity (76%: Pampus et al. 2004), and familiarity with breeding neighbors was found to facilitate cooperative behavior (Grabowska-Zhang et al. 2012a) and increase reproductive output (Grabowska-Zhang et al. 2012b), suggesting that the establishment of long-term social bonds should be under selection in this species. To assess the importance of familiarity in establishing social bonds, we therefore 5) investigate whether familiarity predicts social network stability across seasons for juvenile and adult birds.

## METHODS

### Field methods

**Figure 1:**
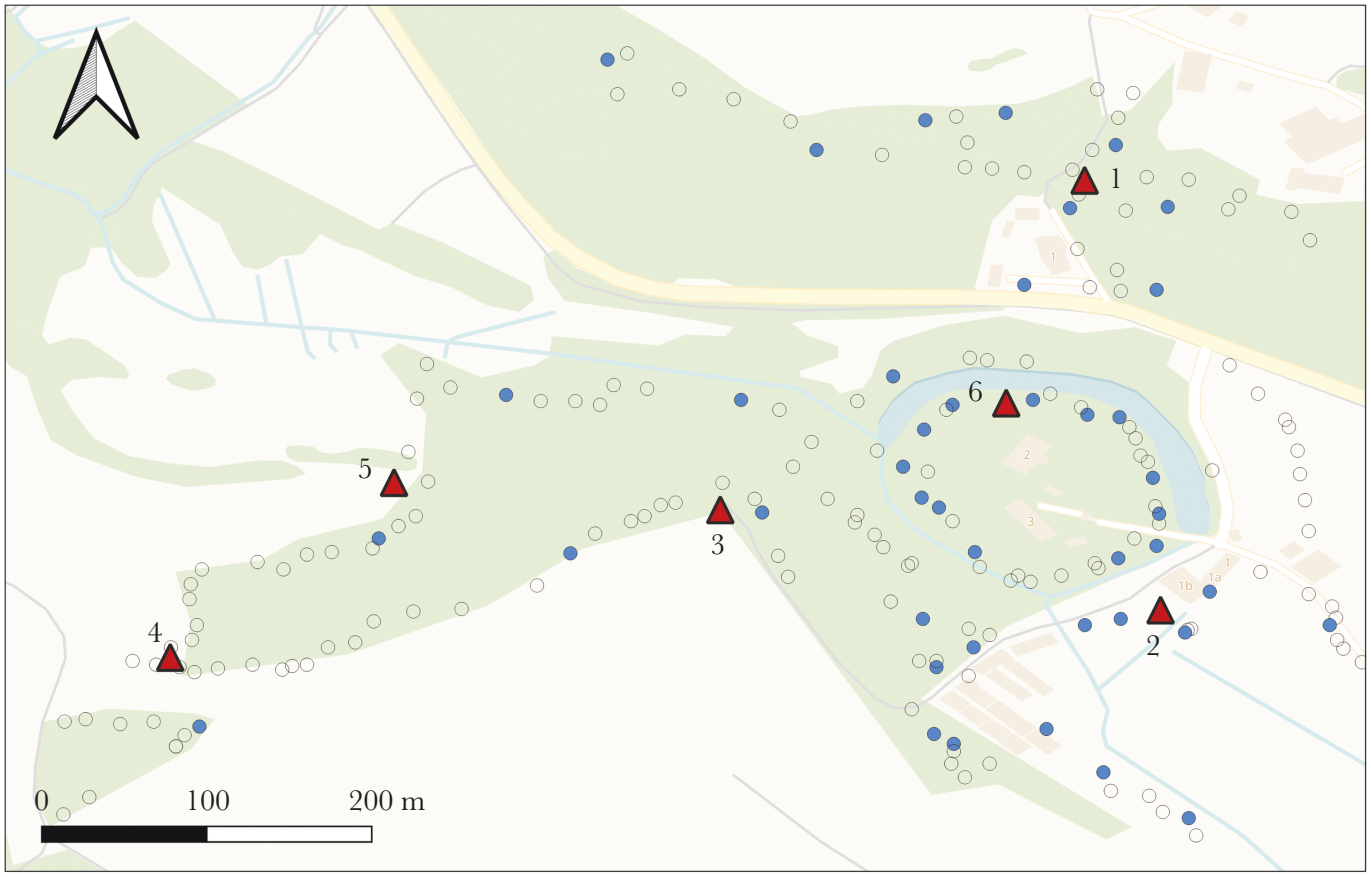
Map of study area. 185 great tit nestlings were PIT-tagged across 43 nest boxes (filled circles; empty circles for other species or unoccupied boxes). Six network feeders equipped with RFID antennae (filled triangles) were then used to track social networks across seasons.

The study took place in the woodland around the Max Planck Institute of Animal Behavior in Radolfzell, Germany (47.76811, 8.99652 [WGS84]) as part of a long-term project on social behavior in wild great tits. Tits were caught in mist nets throughout the year and equipped with a metal leg ring issued by the Radolfzell Bird Observatory and a unique passive integrated transponder (PIT) tag (Eccel Technology, EM4102) for long-term identification, then sexed and aged based on plumage (Svensson 1992). To study their breeding behavior, we provided 207 nest boxes (Schwegler type 1B, 2M, 3SV) across the woodland (Figure 1). During the breeding season (early April until June 2020), nest boxes were monitored at least twice per week, recording nest stages, clutch sizes, start of incubation, and hatch and fledge dates of nestlings. Breeding pairs were identified 4 days after chick hatching with a built-in RFID antenna around the entrance hole of a faceplate logger (Naturecounters Ltd.) that recorded PIT tags of adult birds entering the nest box. Any untagged adult individuals were subsequently caught in the nest box and equipped with a metal leg ring and a PIT tag. On day 15 after hatching, nestlings were temporarily removed from their nest box for weighing and were equipped with a metal leg ring and a PIT tag before being returned to their nest. Note that the sex of nestlings cannot be determined based on plumage (Svensson 1992)—sex was therefore not included in any of the analyses. To determine the exact date of fledging, we returned to the nest daily from 22 days after hatching onwards until all nestlings had fledged.

To record foraging associations among PIT-tagged birds, we deployed six feeders across the woodland at a distance of approximately 150–200 m from each other (Figure 1), each fitted with two RFID antennae to record visiting birds’ PIT tags. The feeders were installed simultaneously for one 48-h period per week during active data collection, following the protocol established in previous studies (Farine et al. 2012). We collected data all throughout the breeding season into summer to record associations of juveniles during the transition to independence (Supplementary Table S1). Furthermore, to investigate social network dynamics throughout birds’ first year of life, we collected data during three consecutive weeks each the following autumn, winter, and spring, with 48 h of data collection in each week (Supplementary Table S1).

### Statistical methods

#### Social networks

We estimated dyadic association strengths based on co-occurrence at network feeders. We first used Gaussian mixture models to locate spatio-temporal clusters in the visitation data (R package “asnipe”), considering the six feeders as separate locations (Psorakis et al. 2012; Farine 2013). From these clusters, we then identified distinct foraging flocks, splitting overlapping groups into distinct flocks using the “gmmevents” function in “asnipe” (Farine 2013). For the initial spring-summer data collection period in 2020, we created a separate social network for each 48-h feeder deployment, resulting in a total of 14 social networks. We subset the weekly spring-summer networks to only include birds that had been detected in a minimum of five distinct groups in each 48-h period. For subsequent seasons (autumn, winter, and following spring) we combined the three 48-h deployments into a single network for each season. For the seasonal networks, we only included birds that had been detected in a minimum of five distinct groups across the three 48-h periods. Following previous work on this species, we created social networks using a gambit of the group approach (Farine 2013; Aplin et al. 2015). We calculated associations based on the simple ratio index, which ranges from 0 if never observed in the same group to 1 if always observed in the same group (Cairns and Schwager 1987; Hoppitt and Farine 2018). All statistical analyses were conducted in R 4.1.2 (R Core Team 2022).

#### Quantifying space use overlap

As individuals can only associate with those they also share space with, it is vital to account for the potential confound between shared space and association patterns, as has been well established in social network analysis (Lusseau et al. 2006; Wey et al. 2008; Farine 2017; Hobson et al. 2021). In all of our analyses, we therefore included a measure of space use overlap for each dyad. In each dyad, one individual was designated as the “focal” individual (see Analysis 1 below). For each focal individual and its associate, we extracted the number of times they had been registered on each of the six network feeders as a proxy of time spent within each feeder area. For each dyad, we then extracted the overlap of time spent in feeder areas as a proportion of the focal individual’s overall time observed, ranging from 0 for no overlap to 1 for full overlap.

#### Analysis 1: Quantifying relationship changes during ontogeny

To investigate how social relationships of juveniles with other birds developed during transition to independence, we ran a Bayesian multi-membership generalized linear mixed effect model (GLMM) on the data collected during late spring and summer. Multi-membership models are statistical models designed to handle nested or hierarchical data (Browne et al. 2001). In social network analysis, they are used to account for the fact that each edge depends on two nodes by including nodes as well as dyads as random effects in the model (e.g., Rushmore et al. 2013; Boyland et al. 2016; Hart et al. 2022). To be able to track changes in dyadic associations from the viewpoint of each juvenile as they increased in age, in each dyad, one individual was designated the “focal” individual and one the non-focal (e.g., see Gerber et al. 2019). To this end, we first extracted the dyadic association strengths of each focal bird (i.e., all juveniles) with all other birds—including other juveniles as well as adults—from the generated weekly social network. We then extracted 1) the type of relationship with the focal (“parents,” “siblings,” “other adults” and “peers”) as a dyadic covariate. Finally, we extracted 2) a measure of space use overlap (for details see above) and 3) the age of the juvenile (measured in days since fledging [centered around 0]), as co-variates specific to the focal individual. Prior to running our model, we controlled for multi-collinearity among predictor variables by calculating the variance inflation factor (“vif”) using the “car” package in R (Bates et al. 2012). In our model, we additionally allowed for an interaction between relationship type and age. Finally, we included the identity of the focal individual as a random effect and the identity of the dyad as a multi-membership random effect in the model (Hart et al. 2022). We fit a zero-inflated beta model (Model 1 in R code) in the R package “brms” (Bürkner 2017), running four chains with 4000 iterations each (2000 for warm-up, 2000 for sampling). We visually inspected trace plots and extracted R-hat values to confirm chain mixture, convergence and stationarity, and conducted a posterior predictive check (McElreath 2020). Finally, we extracted effect sizes and confidence intervals using posterior means and 95% credibility intervals (McElreath 2020). We report odds ratios (OR hereafter) for each predictor, which refer to the change in the odds of an event occurring per one-unit change in the predictor variables, while the other covariates are held constant (Rita and Komonen 2008) (for more details on interpretation of OR, see Supplementary Information). For all GLMM models, we interpreted effects as significant if confidence intervals of OR excluded 1 (i.e., if both lower and upper confidence intervals were either below or above 1).

#### Analysis 2: Flock formation among peers

In a second analysis, we focused on the establishment of associations amongst peers and how these associations changed over time. We investigated whether juveniles preferentially associated with those of similar phenotype—including age similarity and similarity in fledge weight (as a proxy for similarity in developmental stage). As a response variable, we extracted association strengths among peers from each weekly network across the 14 weeks of data collected in spring and summer 2020. In each dyad, each individual was once considered the focal- and once the non-focal individual (see analysis 1).

As predictors, we included 1) the difference in weight in grams (standardized); 2) the age difference in number of days between hatch dates (standardized); and 3) a measure of space use overlap as the focal individual’s proportion of overlap at feeders with the other individual in the dyad (for details on calculation of space use overlap see above). We additionally allowed for interactions between fledge weight and age difference with the focal individual’s age (in days since fledging), to investigate whether homophilic tendencies change with increasing age. ID of the focal individual was included as a random effect and the dyad as a multi-membership random effect (Hart et al. 2022). We ran a zero-inflated Bayesian multi-membership GLMM (Model 2 in R code) with four chains with 6000 iterations (3000 for warm-up, 3000 for sampling) (Bürkner 2017), checking for multi-collinearity and performing model checks and extracting effect sizes as described for analysis 1.

#### Analysis 3: Inheritance of parental social networks

In a third set of models, we investigated a potential inheritance of the parental social network. In the first model (Model 3a), we tested for a direct inheritance of the parental associations by asking whether juveniles were more likely to associate with adult birds that were also associated with their parents. Only dyads with the juvenile as the focal individual were included. In the second model (Model 3b), we tested for a more indirect inheritance of the parental social network by investigating whether associations among peers were predicted by the summed association strengths between their parents. Here, in each dyad, each juvenile was once considered the focal- and once the non-focal individual. To calculate the summed association strengths with (model 3a) and between (model 3b) parents, we always included data from the first week to the current 48-h period (e.g., in week 5, we included data collected in weeks 1–5). For both models, we controlled for space use overlap (for details on space use overlap, see above). We additionally allowed for an interaction between association with/between the parents and the focal individual’s age (in days since fledging) to test whether associations with parental associates or their offspring changed with developmental stage. ID of the focal individual was included as a random effect and the dyad as a multi-membership random effect (Hart et al. 2022). We ran zero-inflated Bayesian multi-membership GLMMs (Models 3a and 3b in R code) with four chains with 6000 iterations (3000 for warm-up, 3000 for sampling) (Bürkner 2017), checking for multi-collinearity and performing model checks and extracting effect sizes as described for analysis 1.

#### Analysis 4: Assortment by age across seasons

In a fourth set of models, we tested for assortment by age among first-year and adult birds. In each season separately (i.e., summer, autumn, winter, spring), we calculated assortativity coefficients (*r*_assort_) by age using package “assortnet” (Farine 2014) (Model 4a-d in R code). These coefficients range from −1 (complete dis-assortment: associations only between but not within age classes) to 1 (complete assortment: associations only within age classes). To calculate the significance of the observed assortativity coefficient (*p*_assort_), we used node-based permutation (Farine 2014). We randomized age for all individuals 1000 times while maintaining the edge structure of the network, and extracted the proportion of randomized values that were larger (for assortment) or smaller (for disassortment) than the observed value. For summer, we used the data collected during the final three weeks of data collection to ensure consistency with other seasons (Supplementary Table S1). Unlike in analyses 1 to 3, we here also included birds that were ringed after the breeding season 2020, that is, had potentially hatched outside of our nest boxes or study area and were later caught in mist nets throughout the year. Hatch year in those cases was assigned based on plumage on the day of capture (Svensson 1992).

#### Analysis 5: Network stability based on familiarity across seasons

In a final set of models, we tested for stability of associations across seasons by testing whether dyadic associations were predicted by familiarity, that is, the dyadic association strengths during the previous season, while controlling for dyadic space use overlap. We additionally allowed for an interaction between association during the previous season and the focal ID’s age (first-year vs. adult birds), to test whether the two age classes differ in their tendency to associate with familiar individuals across seasons. Similarly to analysis 4, we included birds that had been trapped in mist nets throughout the year. We subset the data to birds with dyadic data available in consecutive seasons. We ran three zero-inflated Bayesian multi-membership GLMMs (for autumn, winter, and spring—Models 5a-c in R code) with focal ID and the dyad ID as random factors (Hart et al. 2022) with 6000 iterations each (3000 for warm-up, 3000 for sampling), and performed model checks and extracted effect sizes as described above (see analysis 1).

## RESULTS

We PIT-tagged 185 great tit nestlings in 43 nest boxes (Figure 1), 65 of which were recorded in a minimum of five observations during each data period during summer, 20 during autumn, 30 during winter, and 19 during the subsequent spring. Including all individuals tagged during the breeding season, as well as those tagged throughout the year, a total of 308 unique great tits were recorded on RFID antennae (109 first-year and 90 adults) across all seasons.

### Analysis 1: Quantifying relationship changes during ontogeny

**Figure 2:**
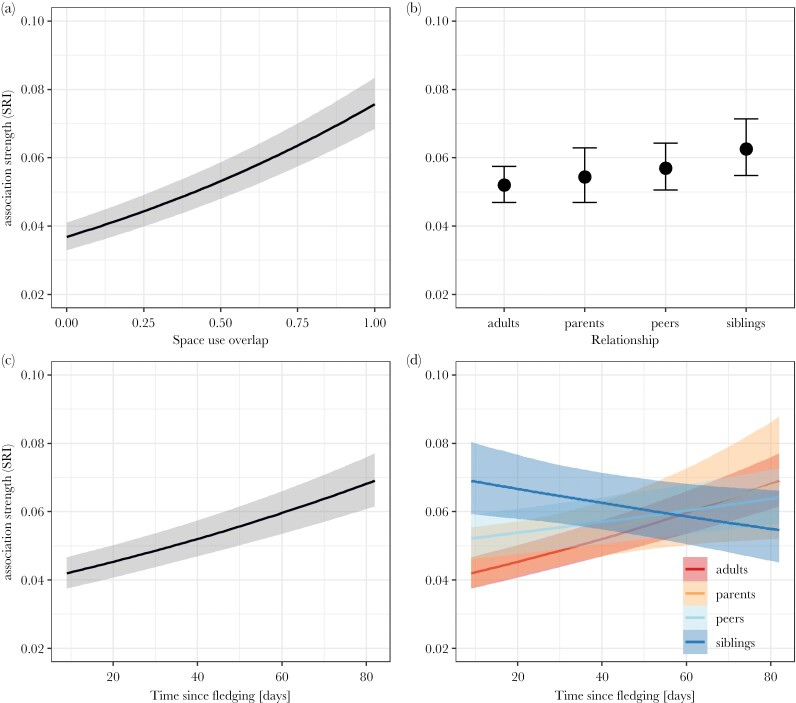
Predicted association strengths of juveniles with other birds during ontogeny including 95% credible intervals. (a) Dyadic association strength predicted by space use overlap. Juveniles were more strongly associated with birds using the same feeder areas. (b) Dyadic association strength predicted by relationship type. Fledglings showed a slight overall preference for associating with siblings and peers over non-parent adults, while there was no evidence for an overall preference for or avoidance of parents. (c) Dyadic association strength predicted by age. Juveniles increased their overall association strength with other birds with increasing age, measured in days since fledging. (d) Change in dyadic association by relationship type with age. While juveniles initially were more strongly associated with siblings over other birds, their dyadic association strengths decreased with increasing age. All other types of relationships (with parents, non-parent adults and peers) increased with increasing age of the juvenile.

In our first analysis on how social associations of juveniles with other birds develop during their transition to independence, we analyzed 17188 dyads of 68 juveniles and 60 adults across a total of 14 weeks of data collection during spring and summer 2020. This only includes dyads where both birds fulfill the inclusion criterion of being present in a minimum of five distinct groups (here and below). We found no evidence for multi-collinearity among predictor variables (Supplementary Table S2). Space use overlap was the strongest predictor of association strength with individuals with increased space use overlap being more likely to associate (odds ratio [OR hereafter]: 2.22; 95% credible interval: [2.09–2.37]; comparing complete to no overlap) (Figure 2a; Supplementary Table S3). Even when controlling for space use overlap, juveniles showed a preference for associating with siblings (OR: 1.23 [1.09–1.39]) and a trend for association with peers (OR: 1.11 [0.99–1.23]) compared to other adults (baseline), while there was no evidence for an overall preference or avoidance of parents (OR: 1.05 [0.93–1.18]; Supplementary Table S3; Figure 2b). Overall, juveniles increased their association strengths to other birds with increasing age (OR: 1.15 [1.12–1.18]; Figure 2c; Supplementary Table S3), with an increase in association strength being strongest between juveniles and non-parent adults (baseline), followed by parent–offspring associations (OR: 0.97 [0.88–1.08]) and those with peers (OR: 0.92 [0.89–0.95]), while dyadic associations with siblings decreased with age (OR: 0.81 [0.76–0.87]; Figure 2d; Supplementary Table S3).

### Analysis 2: Flock formation among non-sibling juveniles

In our second analysis, we examined the factors influencing associations among peers, analyzing 8906 dyads of 65 juveniles across the 14 weeks of data into early summer. We found no evidence for collinearity among predictors (Supplementary Table S2). After controlling for associations being driven by overlap in space use (OR: 2.28 [2.09–2.49], comparing complete to no overlap), we found no evidence for homophilic tendencies (Supplementary Table S4). Juveniles showed no preferences for association with those similar in age (OR: 1.01 [0.98–1.04]; Supplementary Figure S1a), or those of similar fledge weight (OR: 1.01 [0.99–1.04]) (Supplementary Figure S1b). With increasing age, juveniles showed a marginal increase in association strengths with those closer in age, while associations with those more dissimilar in age decreased (OR: 0.95 [9.93–0.98]; Supplementary Figure S1c). Meanwhile, similarity in fledge weight did not predict changes in association strengths over time (OR: 0.99 [0.97–1.01]; Supplementary Figure S1d).

### Analysis 3: Inheritance of parental social networks

For investigating a potential role of an inheritance of the parental social network, we analyzed 7415 dyads of 65 fledglings with 60 non-parent adults and 8906 dyads among 65 juveniles, respectively. After controlling for effects of space use overlap (OR: 2.00 [1.83–2.19]), we found that with increasing age, juveniles were more likely to increase association strengths with other adults, if the adult was also associated with the juvenile’s parents (OR: 2.29 [1.89–2.76]; Supplementary Table S5). Overall, however, the parents’ social network did not predict a juvenile’s associations with non-parent adults (OR: 0.97 [0.65–1.46]; Supplementary Table S5). Similarly, after controlling for effects of similarities in space use (OR: 2.21 [2.02–2.41]), unrelated juveniles whose respective parents were associated were marginally more likely to increase their association strengths with increasing age (OR: 1.24 [1.10–1.39]), but overall, associations among parent pairs did not predict association strengths among their offspring (OR: 1.13 [0.91–1.39]; Supplementary Table S5).

### Analysis 4: Assortment by age across seasons

**Figure 3:**
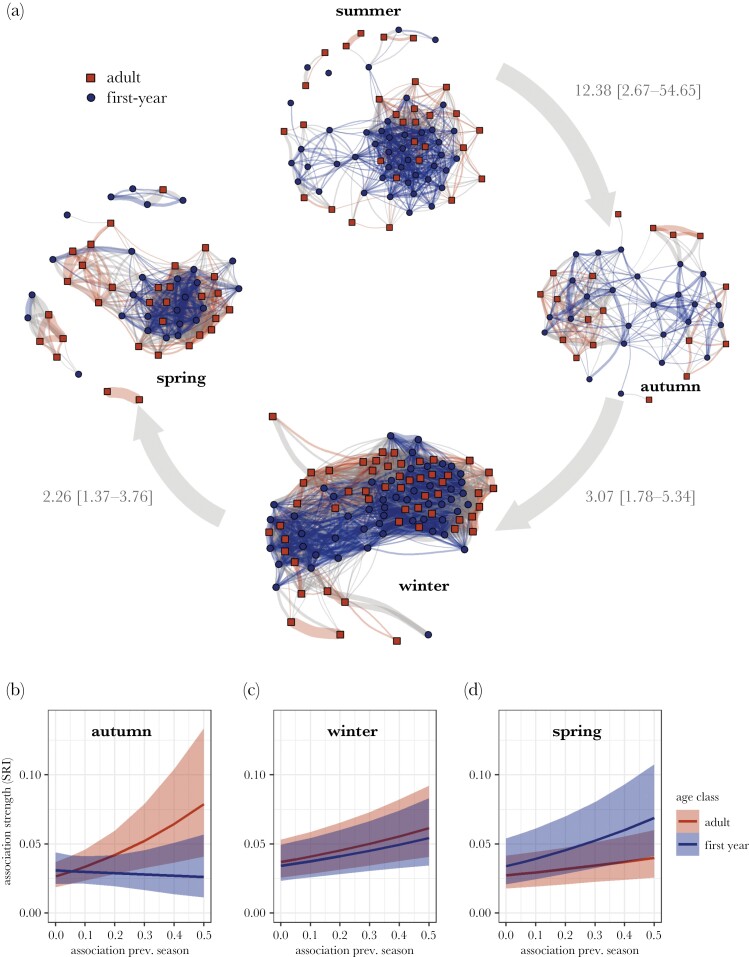
(a) Social networks across seasons. Dyadic associations were predicted by associations during the previous season for both adults and first-year birds (gray arrows indicate odds ratios estimated across both adults and first-years for associations in the previous season predicting associations in the current season (per unit association) with 95% credible intervals). (b–d) Network stability based on familiarity across seasons per age group. Associations during the previous season predicted dyadic association strengths across all seasons. In autumn, this effect was mainly driven by adult birds and in spring by first-year birds, although effect sizes for adults fall largely within credible intervals of first-year birds.

For assessing assortment by age across seasons, we analyzed 5550 dyads during the last three weeks of summer (45 first-year, 30 adult birds), 1806 dyads in autumn (25 first-year, 18 adult birds), 10,920 dyads in winter (52 first-year, 53 adult birds) and 3306 dyads in spring (27 first-year, 31 adult birds). We found no evidence for assortment by age in summer (*r*_assort_ = 0.006; *p*_assort_ = 0.10); winter (*r*_assort_ = −0.005; *p*_assort_ = 0.23), and spring (*r*_assort_ = −0.012; *p*_assort_ = 0.27). Meanwhile, associations in autumn were positively assorted by age (*r*_assort_ = 0.097; *p*_assort_ = 0.03: Figure 3a).

### Analysis 5: Network stability based on familiarity across seasons

In the analysis of whether individuals formed stable social bonds based on previous familiarity, we reduced the data set to dyads that had also been observed during the previous season. This resulted in 870 dyads in autumn (17 first-year, 12 adult birds), 1260 dyads in winter (19 first-year, 16 adult birds), and 2256 dyads in spring (22 first-year, 25 adult birds).

After controlling for effects of space use overlap (OR autumn: 2.70 [1.88–3.92]; OR winter: 14.05 [11.70–16.90]; OR spring: 8.67 [7.23–10.35]), association strengths were strongly predicted by the associations during the previous season across the entire year (OR autumn: 12.38 [2.67–54.65]; OR winter: 3.07 [1.78–5.34]; OR spring: 2.26 [1.37–3.76]; Supplementary Table S6; Figure 3a). Transitioning from summer to autumn, the stability of social associations was stronger in adults compared to first-year birds (OR: 0.06 [0.01–0.64]; Figure 3b), while the stability of associations from autumn to winter was equal for first-years and adult birds (OR: 0.88 [0.38–2.00]; Figure 3c). Transitioning from winter to spring, first-years showed marginally higher stability compared to adults (OR: 2.15 [0.99–4.65]; Supplementary Table S6; Figure 3d).

## DISCUSSION

Songbirds have become a model species for the study of sociality, with over a decade of research exploring the social networks of species such as great tits (Aplin et al. 2014; Snijders et al. 2014), zebra finches (McCowan et al. 2015), house sparrows (Dunning et al. 2023) and golden-crowned sparrows (Arnberg et al. 2015). Yet, most studies to date have focused on associations among birds after reaching full independence (Farine et al. 2012; Aplin et al. 2013; Snijders et al. 2014; Beck et al. 2020), while the ontogeny of sociality among juveniles, including changes in sociality over seasons in the first year of life, have largely been ignored (but see Templeton et al. 2012; Franks et al. 2020). Here, we focus on elucidating the factors contributing to the establishment of social bonds in juvenile great tits during their transition to independence, as well as the formation and stability of networks across different seasons during their first year of life.

First, juveniles increased their dyadic association strengths with other birds over the first few weeks after fledging, indicative of the establishment of a growing social network as they integrated into local flocks upon reaching independence. Consistent with previous work in this species (Farine et al. 2015), both spatial as well as social factors were predictors of dyadic association strength. During this period of growing independence, juveniles showed overall stronger associations with siblings and peers over non-parent adults, while there was no evidence for an overall preference for or avoidance of parents, potentially driven by differences in hatch dates and variation in the duration of dependence periods of juveniles, which can last between 10 and 32 days (Verhulst and Hut 1996). Preferences for associating with similarly aged individuals—also demonstrated in juvenile hihis (Franks et al. 2020)—can provide benefits if those individuals hold social information that is most relevant for their current developmental needs. In fact, peers have been shown to be important sources of social information across taxa (Templeton et al. 2012; Gallois et al. 2018), highlighting the importance of potential horizontal information transfer during development in species with limited periods of parental care. Such horizontal transmission is likely facilitated by higher social tolerance among juvenile individuals, as has been observed in other species (e.g., vervet monkey [*Chlorocebus pygerythrus*]: Grampp et al. 2019). Alternatively, preferences for associating with other juveniles may be driven by the risk of aggression from non-parent adults (Templeton et al. 2012; Krzyszczyk et al. 2017).

Within this overall preference for peers and those close in space, we found no evidence for homophilic tendencies among non-sibling juveniles based on our measured traits. After controlling for overlap in space use, we found no preference for associations among juveniles close in age or similar in fledge weight, which we used as a proxy for body condition. Interestingly, we found a marginal increase in associations among juveniles as they increased in age if their respective parents were associated compared to those whose parents were not associated, indicative of a potential influence of inheritance of the parents’ social network. Similarly, juveniles showed a weak preference for increasing associations with their parents’ adult associates over time. Overall, however, the parental social network did not predict their offspring’s social connections. Inheritance of social networks has been demonstrated in several species, including spotted hyenas (*Crocuta crocuta*) (Ilany et al. 2020) and chacma baboons (*Papio ursinus*) (Roatti et al. 2023). In the great tits, however, the effect was small, indicating that juveniles may prioritize other (unmeasured) criteria for establishing their preferred associations, such as personality traits, which have been demonstrated to influence both structure and temporal stability of networks among fully independent individuals in this species (Aplin et al. 2013; Snijders et al. 2014; Johnson et al. 2017).

The period of preferential association among juveniles was temporary, with the initial preference for associating with peers and siblings decreasing during a juvenile’s first few weeks after fledging and association strengths with adults increasing. These findings were supported by a lack of evidence for assortment by age during the final 3 weeks of summer data, suggesting that by this time, first-year birds had already fully integrated into local flocks of adult birds. Yet, we found some evidence for positive assortment by age in autumn, with first-year birds showing a weak preference for associating with other first-year birds over adults and vice versa. Furthermore, while association patterns during summer were a predictor of associations during autumn in adults, this was observed to a lesser extent in first-year birds. These observations could be driven by demographic processes, as great tits exhibit (female-biased) dispersal during the first three months after reaching independence (Greenwood and Harvey 1982; Dingemanse et al. 2003; Michler et al. 2011), coinciding with the transition from summer to autumn in our data. Since the sex of juveniles cannot yet be determined based on plumage before the first molt (Svensson 1992), we were unable to determine whether the lack of stability in the association among juveniles was mostly driven by females, as expected in a system with female-biased dispersal.

Yet, overall, dyadic association strengths were predicted by the association strengths during the previous season, indicating that individuals established long-term stable bonds with already familiar individuals. Familiarity has been shown to be a strong driver of sociality across various taxa (Griffiths and Magurran 1999; King et al. 2011; Kohn et al. 2015; Keller et al. 2017) and can provide mutual direct fitness benefits through reduced aggression (Senar et al. 1990; Temeles 1994), facilitate the discovery and sharing of resources (Carter and Wilkinson 2013; Atton et al. 2014), and promote cooperative behavior (Gerber et al. 2021). Associating with familiar individuals has been further shown to positively influence survival (Silk et al. 2010) and reproduction (Cameron et al. 2009; Riehl and Strong 2018).

In great tits, familiarity among breeding neighbors was found to lead to increased rates of joint nest defense behavior (Grabowska-Zhang et al. 2012) and increased reproductive output (Grabowska-Zhang et al. 2012). Long-term stable bonds are therefore expected to be under positive selection in this species, and this prediction is further supported by our results. Interestingly, first-year birds exhibited higher social stability from winter to spring than adults. Great tits are territorial while breeding, which dramatically changes their social network. Yet, while we did not specifically examine this in our analysis, in our population, individuals often do not appear to gain breeding opportunities until two years of age. It is possible that this age-related difference in stability of associations, therefore, reflects an extension of winter flocking behavior into the spring by non-breeding juveniles.

Taken together, we found that the social networks of juveniles undergo dynamic changes during transition to independence with associations being shaped by both spatial and social factors, which most likely reflect changes in selective pressures during development. By their first summer, first-year birds were fully integrated into the local flocks. By autumn, they established social strategies similar to those of adults by forming stable social ties based on familiarity, which persisted through winter and into spring. These findings align with and extend previous research in this species, which found that great tits form social bonds that carry over across behavioral contexts (Firth and Sheldon 2015) and time (Firth and Sheldon 2016), with profound consequences for many biological processes including reproduction (Grabowska-Zhang et al. 2012; Grabowska-Zhang et al. 2012; Farine and Sheldon 2015) and transmission of social information (Aplin et al. 2012; Firth et al. 2016).

Age-driven changes in social network stability and network characteristics have been demonstrated in a diverse set of other species, including giraffes (*Giraffa camelopardalis*) (Muller et al. 2022), African elephants (Murphy et al. 2020), vervet monkeys (Borgeaud et al. 2017), sulphur-crested cockatoos (*Cacatua galerita*) (Aplin et al. 2021), chimpanzees (*Pan troglodytes*) (Thompson González et al. 2021) and rhesus macaques (*Macaca mulatta*) (Liao et al. 2018; Siracusa et al. 2022). Given the slow life history in those species, significant changes in sociality are usually observed over the period of several months or years. Our study provides a contrast in great tits, where dynamic changes in sociality occur on a much faster temporal scale of several weeks, and where social stability is reached within months of reaching independence, reflecting the fast life history and a short period of parental care in this species. Yet, while brief, this period of initial independence is also a target of intense selection, for which social networks are likely to be vital. Future work should continue to examine the causes and consequences of variation in social networks during this time and further establish the role of variation in the length of post-fledge care (Franks et al. 2019) and demographic processes such as (sex-specific) dispersal on the formation and stability of social networks (Shizuka and Johnson 2020).

## Supplementary Material

arae011_suppl_Supplementary_Material

## Data Availability

Analyses reported in this article can be reproduced using the data provided by Wild et al. (2024).

## References

[CIT0001] Almeling L , Hammerschmidt K, Sennhenn-Reulen H, Freund AM, Fischer J. 2016. Motivational shifts in aging monkeys and the origins of social selectivity. Curr Biol. 26(13):1744–1749. doi:10.1016/j.cub.2016.04.066 27345168

[CIT0002] Aplin LM , Farine DR, Mann RP, Sheldon BC. 2014. Individual-level personality influences social foraging and collective behaviour in wild birds. Proc R Soc B: Biol Sci. 281(20141016):20141016. 10.1098/rspb.2014.1016 PMC4100518 24990682

[CIT0003] Aplin LM , Farine DR, Morand-Ferron J, Cockburn A, Thornton A, Sheldon BC. 2015. Experimentally induced innovations lead to persistent culture via conformity in wild birds. Nature. 518:538–541. doi:10.1038/nature13998. http://www.nature.com/doifinder/10.1038/nature13998 25470065 PMC4344839

[CIT0004] Aplin LM , Farine DR, Morand-Ferron J, Cole EF, Cockburn A, Sheldon BC. 2013. Individual personalities predict social behaviour in wild networks of great tits (*Parus major*). Ecol Lett. 16(11):1365–1372. doi:10.1111/ele.12181 24047530

[CIT0005] Aplin LM , Farine DR, Morand-Ferron J, Sheldon BC. 2012. Social networks predict patch discovery in a wild population of songbirds. Proceedings of the Royal Society B. 279(1745):4199–4205. doi:10.1098/rspb.2012.1591 22915668 PMC3441092

[CIT0006] Aplin LM , Major RE, Davis A, Martin JM. 2021. A citizen science approach reveals long-term social network structure in an urban parrot, *Cacatua galerita*. J Anim Ecol. 90(1):222–232. doi:10.1111/1365-2656.13295 32629533

[CIT0007] Arnberg NN , Shizuka D, Chaine AS, Lyon BE. 2015. Social network structure in wintering golden-crowned sparrows is not correlated with kinship. Mol Ecol. 24(19):5034–5044. doi:10.1111/mec.13366 26334186

[CIT0008] Atton N , Galef BJ, Hoppitt W, Webster MM, Laland KN. 2014. Familiarity affects social network structure and discovery of prey patch locations in foraging stickleback shoals. Proc R Soc B: Biol Sci. 281(1789):20140579. doi:10.1098/rspb.2014.0579 PMC4100505 25009061

[CIT0009] Bates D , Firth D, Friendly M, Gorjanc G, Graves S, Heiberger R, Monette G, Nilsson H, Ripley B, Weisberg S, et al. 2012. Package 'car'. *Vienna: R Foundation for Statistical Computing*, 16.

[CIT0010] Beck KB , Farine DR, Kempenaers B. 2020. Winter associations predict social and extra-pair mating patterns in a wild songbird. Proc R Soc B: Biol Sci. 287(1921):20192606. doi:10.1098/rspb.2019.2606 PMC7062020 32070248

[CIT0011] Bizzozzero MR , Allen SJ, Gerber L, Wild S, King SL, Connor RC, Friedman WR, Wittwer S, Krützen M. 2019. Tool use and social homophily among male bottlenose dolphins. Proc R Soc B: Biol Sci. 286(20190898):20190898. 10.1098/rspb.2019.0898 PMC6571475 31185859

[CIT0012] Blonder B , Wey TW, Dornhaus A, James R, Sih A. 2012. Temporal dynamics and network analysis. Methods Ecol Evol. 3(6):958–972. doi:10.1111/j.2041-210X.2012.00236.x

[CIT0013] Borgeaud C , Sosa S, Sueur C, Bshary R. 2017. The influence of demographic variation on social network stability in wild vervet monkeys. Anim Behav. 134:155–165. doi:10.1016/j.anbehav.2017.09.028

[CIT0014] Boyland NK , Mlynski DT, James R, Brent LJN, Croft DP. 2016. The social network structure of a dynamic group of dairy cows: from individual to group level patterns. Appl Anim Behav Sci. 174:1–10. doi:10.1016/j.applanim.2015.11.016

[CIT0015] Browne WJ , Goldstein H, Rasbash J. 2001. Multiple membership multiple classiﬁcation (MMMC) models. Stat Modelling. 1:103–124.

[CIT0016] Bull CM , Godfrey SS, Gordon DM. 2012. Social networks and the spread of Salmonella in a sleepy lizard population. Mol Ecol. 21(17):4386–4392. doi:10.1111/j.1365-294X.2012.05653.x 22845647

[CIT0017] Bürkner PC. 2017. brms: an R package for Bayesian multilevel models using Stan. J Stat Softw. 80:1–28. doi:10.18637/jss.v080.i01

[CIT0018] Cairns SJ , Schwager SJ. 1987. A comparison of association indices. Anim Behav. 35(5):1454–1469.

[CIT0019] Cameron EZ , Setsaas TH, Linklater WL. 2009. Social bonds between unrelated females increase reproductive success in feral horses. Proc Natl Acad Sci USA. 106(33):13850–13853. https://www.pnas.org 19667179 10.1073/pnas.0900639106 PMC2728983

[CIT0020] Carter AJ , Lee AEG, Marshall HH, Ticó MT, Cowlishaw G. 2015. Phenotypic assortment in wild primate networks: implications for the dissemination of information. R Soc Open Sci. 2(5):140444. doi:10.1098/rsos.140444 26064652 PMC4453262

[CIT0021] Carter GG , Wilkinson GS. 2013. Food sharing in vampire bats: Reciprocal help predicts donations more than relatedness or harassment. Proc Royal Soc B: Biol Sci. 280(1753):20122573. doi:10.1098/rspb.2012.2573 PMC3574350 23282995

[CIT0022] Carter KD , Brand R, Carter JK, Shorrocks B, Goldizen AW. 2013. Social networks, long-term associations and age-related sociability of wild giraffes. Anim Behav. 86(5):901–910. doi:10.1016/j.anbehav.2013.08.002

[CIT0023] Carter KD , Seddon JM, Frère CH, Carter JK, Goldizen AW. 2013. Fission-fusion dynamics in wild giraffes may be driven by kinship, spatial overlap and individual social preferences. Anim Behav. 85(2):385–394. doi:10.1016/j.anbehav.2012.11.011

[CIT0024] Chiyo PI , Archie EA, Hollister-Smith JA, Lee PC, Poole JH, Moss CJ, Alberts SC. 2011. Association patterns of African elephants in all-male groups: the role of age and genetic relatedness. Anim Behav. 81(6):1093–1099. doi:10.1016/j.anbehav.2011.02.013

[CIT0025] Darden SK , James R, Ramnarine IW, Croft DP. 2009. Social implications of the battle of the sexes: sexual harassment disrupts female sociality and social recognition. Proc Biol Sci. 276(1667):2651–2656. doi:10.1098/rspb.2009.0087 19386653 PMC2686652

[CIT0026] de Lima VCC , Ferreira RG. 2021. Social network changes during the development of immature capuchin monkeys (Sapajus spp.). Primates. 62:801–815. doi:10.1007/s10329-021-00918-6 34273030

[CIT0027] Diaz-Aguirre F , Parra GJ, Passadore C, Möller L. 2018. Kinship influences social bonds among male southern Australian bottlenose dolphins (*Tursiops cf. australis*). Behav Ecol Sociobiol. 72(12):1–13. doi:10.1007/s00265-018-2621-4

[CIT0028] Dingemanse NJ , Both C, Van Noordwijk AJ, Rutten AL, Drent PJ. 2003. Natal dispersal and personalities in great tits (Parus major). Proc Biol Sci. 270(1516):741–747. doi:10.1098/rspb.2002.2300 12713749 PMC1691302

[CIT0029] Dunning J , Burke T, Hoi Hang Chan A, Ying Janet Chik H, Evans T, Schroeder J. 2023. Opposite-sex associations are linked with annual fitness, but sociality is stable over lifetime. Behav Ecol. 34(3):315–324. doi:10.1093/beheco/arac124 37192923 PMC10183206

[CIT0030] Farine DR. 2013. Animal social network inference and permutations for ecologists in R using asnipe. Methods Ecol Evol. 4(12):1187–1194. doi:10.1111/2041-210X.12121

[CIT0031] Farine DR. 2014. Measuring phenotypic assortment in animal social networks: weighted associations are more robust than binary edges. Anim Behav. 89:141–153. doi:10.1016/j.anbehav.2014.01.001

[CIT0032] Farine DR. 2017. A guide to null models for animal social network analysis. Methods Ecol Evol. 8(10):1309–1320. doi:10.1111/2041-210X.12772 29104749 PMC5656331

[CIT0033] Farine DR , Aplin LM, Sheldon BC, Hoppitt W. 2015. Interspecific social networks promote information transmission in wild songbirds. Proc Biol Sci. 282(1803):20142804. doi:10.1098/rspb.2014.2804. 25673683 PMC4345451

[CIT0034] Farine DR , Firth JA, Aplin LM, Crates RA, Culina A, Garroway CJ, Hinde CA, Kidd LR, Milligan ND, Psorakis I, et al. 2015. The role of social and ecological processes in structuring animal populations: a case study from automated tracking of wild birds. R Soc Open Sci. 2(4):150057–150057. doi:10.1098/rsos.150057 26064644 PMC4448873

[CIT0035] Farine DR , Garroway CJ, Sheldon BC. 2012. Social network analysis of mixed-species flocks: exploring the structure and evolution of interspecific social behaviour. Anim Behav. 84(5):1271–1277. doi:10.1016/j.anbehav.2012.08.008

[CIT0036] Farine DR , Sheldon BC. 2015. Selection for territory acquisition is modulated by social network structure in a wild songbird. J Evol Biol. 28(3):547–556. doi:10.1111/jeb.12587 25611344 PMC4406129

[CIT0037] Firth JA , Sheldon BC. 2015. Experimental manipulation of avian social structure reveals segregation is carried over across contexts. Proc R Soc B: Biol Sci. 282(1802):20142350. doi:10.1098/rspb.2014.2350 PMC4344146 25652839

[CIT0038] Firth JA , Sheldon BC. 2016. Social carry-over effects underpin trans-seasonally linked structure in a wild bird population. Ecol Lett. 19(11):1324–1332. doi:10.1111/ele.12669 27623746 PMC5082527

[CIT0039] Firth JA , Sheldon BC, Farine DR, Firth JA, Farine DR. 2016. Pathways of information transmission among wild songbirds follow experimentally imposed changes in social foraging structure. Biol Lett. 12(20160144):1–4. doi:10.1098/rsbl.2016.0144. PMC4938043 27247439

[CIT0040] Franks VR , Ewen JG, McCready M, Rowcliffe JM, Smith D, Thorogood R. 2020. Analysing age structure, residency and relatedness uncovers social network structure in aggregations of young birds. Anim Behav. 166:73–84. doi:10.1016/j.anbehav.2020.06.005

[CIT0041] Franks VR , McCready M, Savage JL, Thorogood R. 2019. Time spent with parents varies with early-life condition, but does not predict survival or sociality of juvenile hihi. Front Ecol Evol. 7(AUG):1–8. doi:10.3389/fevo.2019.00322

[CIT0042] Gallois S , Lubbers MJ, Hewlett B, Reyes-García V. 2018. Social networks and knowledge transmission strategies among baka children, southeastern cameroon. Human Nature. 29:442–463. doi:10.1007/s12110-018-9328-0 30357606 PMC6208833

[CIT0043] Gerber L , Connor RC, King SL, Allen SJ, Wittwer S, Bizzozzero MR, Friedman WR, Kalberer S, Sherwin WB, Wild S, et al. 2019. Affiliation history and age similarity predict alliance formation in adult male bottlenose dolphins. Behav Ecol. 31:361–370. doi:10.1093/beheco/arz195 32210525 PMC7083095

[CIT0044] Gerber L , Wittwer S, Allen SJ, Holmes KG, King SL, Sherwin WB, Wild S, Willems EP, Connor RC, Krützen M. 2021. Cooperative partner choice in multi-level male dolphin alliances. Sci Rep. 11(1):6901. doi:10.1038/s41598-021-85583-x. http://www.nature.com/articles/s41598-021-85583-x 33767258 PMC7994371

[CIT0045] Gerlach G , Hodgins-Davis A, MacDonald B, Hannah RC. 2007. Benefits of kin association: related and familiar zebrafish larvae (*Danio rerio*) show improved growth. Behav Ecol Sociobiol. 61(11):1765–1770. doi:10.1007/s00265-007-0409-z

[CIT0046] Goldenberg SZ , Douglas-Hamilton I, Wittemyer G. 2016. Vertical transmission of social roles drives resilience to poaching in elephant networks. Curr Biol. 26(1):75–79. doi:10.1016/j.cub.2015.11.005 26711491

[CIT0047] Grabowska-Zhang AM , Sheldon BC, Hinde CA. 2012a. Long-term familiarity promotes joining in neighbour nest defence. Biol Lett. 8(4):544–546. doi:10.1098/rsbl.2012.0183 22535641 PMC3391480

[CIT0048] Grabowska-Zhang AM , Wilkin TA, Sheldon BC. 2012b. Effects of neighbor familiarity on reproductive success in the great tit (*Parus major*). Behav Ecol. 23(2):322–333. doi:10.1093/beheco/arr189

[CIT0049] Grampp M , Sueur C, van de Waal E, Botting J. 2019. Social attention biases in juvenile wild vervet monkeys: implications for socialisation and social learning processes. Primates. 60:261–275. doi:10.1007/s10329-019-00721-4 30941537

[CIT0050] Greenwood PJ , Harvey PH. 1982. The Natal and Breeding Dispersal of Birds. https://www.jstor.org/stable/2097060?seq=1&cid=pdf-

[CIT0051] Griffiths SW , Magurran AE. 1999. Schooling decisions in guppies (*Poecilia reticulata*) are based on familiarity rather than kin recognition by phenotype matching. Behav Ecol Sociobiol. 45:437–443.

[CIT0052] Hamede RK , Bashford J, McCallum H, Jones M. 2009. Contact networks in a wild Tasmanian devil (*Sarcophilus harrisii*) population: using social network analysis to reveal seasonal variability in social behaviour and its implications for transmission of devil facial tumour disease. Ecol Lett. 12(11):1147–1157. doi:10.1111/j.1461-0248.2009.01370.x 19694783

[CIT0053] Hart JDA , Weiss MN, Brent LJN, Franks DW. 2022. Common permutation methods in animal social network analysis do not control for non-independence. Behav Ecol Sociobiol. 76(151):151. doi:10.1101/2021.06.04.447124 36325506 PMC9617964

[CIT0054] Hobson EA , Silk MJ, Fefferman NH, Larremore DB, Rombach P, Shai S, Pinter‐Wollman N. 2021. A guide to choosing and implementing reference models for social network analysis. Biol Rev. 96(6):2716–2734. doi:10.1111/brv.12775. https://onlinelibrary.wiley.com/doi/10.1111/brv.12775 34216192 PMC9292850

[CIT0055] Hoppitt W , Farine D. 2018. Association indices for quantifying social relationships: how to deal with missing observations of individuals or groups. Anim Behav. 136:227–238. doi:10.1016/j.anbehav.2017.08.029

[CIT0056] Ilany A , Akçay E. 2016. Social inheritance can explain the structure of animal social networks. Nat Commun. 7(12084):12084. doi:10.1038/ncomms12084 27352101 PMC4931239

[CIT0057] Ilany A , Holekamp K, Akçay E. 2020. Rank-dependent social inheritance determines social network structure in a wild mammal population. *Science.* 373(6552):348–352. doi:10.1101/2020.04.10.036087 34437155

[CIT0058] Johnson KVA , Aplin LM, Cole EF, Farine DR, Firth JA, Patrick SC, Sheldon BC. 2017. Male great tits assort by personality during the breeding season. Anim Behav. 128:21–32. doi:10.1016/j.anbehav.2017.04.001 28669996 PMC5478380

[CIT0059] Jones TB , Aplin LM, Devost I, Morand-Ferron J. 2017. Individual and ecological determinants of social information transmission in the wild. Anim Behav. 129:93–101. doi:10.1016/j.anbehav.2017.05.011.

[CIT0060] Keller BA , Finger JS, Gruber SH, Abel DC, Guttridge TL. 2017. The effects of familiarity on the social interactions of juvenile lemon sharks, *Negaprion brevirostris*. J Exp Mar Biol Ecol. 489:24–31. doi:10.1016/j.jembe.2017.01.004

[CIT0061] Kelley JL , Morrell LJ, Inskip C, Krause J, Croft DP. 2011. Predation risk shapes social networks in fission-fusion populations. PLoS One. 6(8):e24280. doi:10.1371/journal.pone.0024280 21912627 PMC3166168

[CIT0062] King AJ , Clark FE, Cowlishaw G. 2011. The dining etiquette of desert baboons: the roles of social bonds, kinship, and dominance in co-feeding networks. Am J Primatol. 73(8):768–774. doi:10.1002/ajp.20918 21246590

[CIT0063] Kluijver HN. 1951. The population ecology of the great tit, Parus m. major L. *Ardea*. 39:1–135.

[CIT0064] Kohn GM , Meredith GR, Magdaleno FR, King AP, West MJ. 2015. Sex differences in familiarity preferences within fission-fusion brown-headed cowbird, Molothrus ater, flocks. Anim Behav. 106:137–143. doi:10.1016/j.anbehav.2015.05.023

[CIT0065] Krzyszczyk E , Patterson EM, Stanton MA, Mann J. 2017. The transition to independence: sex differences in social and behavioural development of wild bottlenose dolphins. Anim Behav. 129:43–59. doi:10.1016/j.anbehav.2017.04.011

[CIT0066] Langley EJG , van Horik JO, Whiteside MA, Beardsworth CE, Weiss MN, Madden JR. 2020. Early-life learning ability predicts adult social structure, with potential implications for fitness outcomes in the wild. J Anim Ecol. 89(6):1340–1349. doi:10.1111/1365-2656.13194 32118295

[CIT0067] Leu ST , Farine DR, Wey TW, Sih A, Bull CM. 2016. Environment modulates population social structure: experimental evidence from replicated social networks of wild lizards. Anim Behav. 111:23–31. doi:10.1016/j.anbehav.2015.10.001

[CIT0068] Liao Z , Sosa S, Wu C, Zhang P. 2018. The influence of age on wild rhesus macaques’ affiliative social interactions. Am J Primatol. 80(2):1–10. doi:10.1002/ajp.22733 29266298

[CIT0069] Lusseau D , Wilson B, Hammond PS, Grellier K, Durban JW, Parsons KM, Barton TR, Thompson PM. 2006. Quantifying the influence of sociality on population structure in bottlenose dolphins. J Anim Ecol. 75(1):14–24. doi:10.1111/j.1365-2656.2005.01013.x 16903039

[CIT0070] Massen JJM , Koski SE. 2014. Chimps of a feather sit together: chimpanzee friendships are based on homophily in personality. Evol Hum Behav. 35(1):1–8. doi:10.1016/j.evolhumbehav.2013.08.008

[CIT0071] McCowan LSC , Mainwaring MC, Prior NH, Griffith SC. 2015. Personality in the wild zebra finch: exploration, sociality, and reproduction. Behav Ecol. 26(3):735–746. doi:10.1093/beheco/aru239

[CIT0072] McElreath R. 2020. Statistical rethinking: A Bayesian cuorse with examples in R and Stan, second edition. Chapman and Hall/CRC. doi:10.1201/9780429029608

[CIT0073] Michler SPM , Nicolaus M, Ubels R, van der Velde M, Komdeur J, Both C, Tinbergen JM. 2011. Sex-specific effects of the local social environment on juvenile post-fledging dispersal in great tits. Behav Ecol Sociobiol. 65(10):1975–1986. doi:10.1007/s00265-011-1207-1 21957327 PMC3172419

[CIT0074] Muller Z , Cuthill IC, Harris S. 2022. Adolescence and the development of social behaviour in giraffes. Mamm Biol. 102:1333–1343. doi:10.1007/s42991-021-00197-0. https://link.springer.com/10.1007/s42991-021-00197-0

[CIT0075] Murphy D , Mumby HS, Henley MD. 2020. Age differences in the temporal stability of a male African elephant (*Loxodonta africana*) social network. Behav Ecol. 31(1):21–31. doi:10.1093/beheco/arz152

[CIT0076] Naef-Daenzer B , Widmer F, Nuber M. 2001. Differential post-fledging survival of great and coal tits in relation to their condition and fledging date. J Anim Ecol. 70(5):730–738. doi:10.1046/j.0021-8790.2001.00533.x

[CIT0077] Pampus M , Schmidt K-H, Wiltschko W. 2004. Pair bond and breeding success in Blue Tits *Parus caeruleus* and Great Tits *Parus major*. Ibis. 147(1):92–108. doi:10.1111/j.1474-919x.2004.00376.x

[CIT0078] Perrins CM. 1979. British tits. Collins, London. New Naturalist No. 62.

[CIT0079] Psorakis I , Roberts SJ, Rezek I, Sheldon BC. 2012. Inferring social network structure in ecological systems from spatiotemporal data streams. J R Soc Interface. 9(76):3055–3066. doi:10.1098/rsif.2012.0223 22696481 PMC3479900

[CIT0080] Puehringer-Sturmayr V , Stiefel T, Kotrschal K, Kleindorfer S, Frigerio D. 2021. Social interactions change with season and age in Northern Bald Ibis. J Ornithol. 162(1):277–288. doi:10.1007/s10336-020-01824-2

[CIT0081] R Core Team. 2022. R: A language and environment for statistical computing. https://www.r-project.org/

[CIT0082] Riehl C , Strong MJ. 2018. Stable social relationships between unrelated females increase individual fitness in a cooperative bird. Proc R Soc B: Biol Sci. 285(1876):20180130. doi:10.1098/rspb.2018.0130 PMC5904317 29643212

[CIT0083] Rita H , Komonen A. 2008. Odds ratio: an ecologically sound tool to compare proportions. Ann Zool Fennici. 45(1):66–72. doi:10.5735/086.045.0106

[CIT0084] Roatti V , Cowlishaw G, Huchard E, Carter A. 2023. Social network inheritance and differentiation in wild baboons. R Soc Open Sci. 10(5):230219. doi:10.1098/rsos.230219 37234491 PMC10206475

[CIT0085] Rosati AG , Hagberg L, Enigk DK, Otali E, Thompson ME, Muller MN, Wrangham RW, Machanda ZP. 2020. Social selectivity in aging wild chimpanzees. Science (1979). 370(6515):473–476. 10.1126/science.aaz9129 PMC7668794 33093111

[CIT0086] Rushmore J , Caillaud D, Matamba L, Stumpf RM, Borgatti SP, Altizer S. 2013. Social network analysis of wild chimpanzees provides insights for predicting infectious disease risk. J Anim Ecol. 82(5):976–986. doi:10.1111/1365-2656.12088 23734782

[CIT0087] Schoepf I , Schradin C. 2012. Better off alone! Reproductive competition and ecological constraints determine sociality in the African striped mouse (*Rhabdomys pumilio*). J Anim Ecol. 81(3):649–656. 22220746 10.1111/j.1365-2656.2011.01939.x

[CIT0088] Schülke O , Bhagavatula J, Vigilant L, Ostner J. 2010. Social bonds enhance reproductive success in male macaques. Curr Biol. 20(24):2207–2210. doi:10.1016/j.cub.2010.10.058 21093261

[CIT0089] Senar JC , Camerino M, Metcalfe NB. 1990. Familiarity breeds tolerance: the development of social stability in flocking siskins (*Carduelis spinus*). Ethology. 85(1):13–24. doi:10.1111/j.1439-0310.1990.tb00381.x

[CIT0090] Shizuka D , Johnson AE. 2020. How demographic processes shape animal social networks. Behav Ecol. 31(1):1–11. doi:10.1093/beheco/arz083

[CIT0091] Silk JB. 2007. The adaptive value of sociality in mammalian groups. Philosophical Transactions of the Royal Society B: Biological Sciences. 362(1480):539–559. doi: 10.1098/rstb.2006.1994 PMC2346516 17363359

[CIT0092] Silk JB , Beehner JC, Bergman TJ, Crockford C, Engh AL, Moscovice LR, Wittig RM, Seyfarth RM, Cheney DL. 2010. Strong and consistent social bonds enhance the longevity of female baboons. Curr Biol. 20(15):1359–1361. doi:10.1016/j.cub.2010.05.067 20598541

[CIT0093] Siracusa ER , Negron-Del Valle JE, Phillips D, Platt ML, Higham JP, Snyder-Mackler N, Brent LJN. 2022. Within-individual changes reveal increasing social selectivity with age in rhesus macaques. Proc Natl Acad Sci USA. 119(49):e2209180119. doi:10.1073/pnas.2209180119 36445967 PMC9894112

[CIT0094] Snijders L , van Rooij EP, Burt JM, Hinde CA, van Oers K, Naguib M. 2014. Social networking in territorial great tits: slow explorers have the least central social network positions. Anim Behav. 98:95–102. doi:10.1016/j.anbehav.2014.09.029

[CIT0095] Stanton MA , Mann J. 2012. Early social networks predict survival in wild bottlenose dolphins. PLoS One. 7(10):e47508. doi:10.1371/journal.pone.0047508 23077627 PMC3471847

[CIT0096] Svensson L. 1992. Identiﬁcation guide to European passerines. Stockholm: Lars Svensson.

[CIT0097] Temeles EJ. 1994. The role of neighbours in territorial systems: when are they “dear enemies?”. Anim Behav. 47(2):339–350.

[CIT0098] Templeton CN , Reed VA, Campbell SE, Beecher MD. 2012. Spatial movements and social networks in juvenile male song sparrows. Behav Ecol. 23(1):141–152. doi:10.1093/beheco/arr167 22479140 PMC3242974

[CIT0099] Thompson González N , Machanda Z, Otali E, Muller MN, Enigk DK, Wrangham R, Emery Thompson M. 2021. Age-related change in adult chimpanzee social network integration. Evol Med Public Health. 9(1):448–459. doi:10.1093/emph/eoab040 34987824 PMC8697844

[CIT0100] Verhulst S , Hut RA. 1996. Post-fledging care, multiple breeding and the costs of reproduction in the great tit. Anim Behav. 51(5):957–966. doi:10.1006/anbe.1996.0099

[CIT0101] Welklin JF , Lantz SM, Khalil S, Moody NM, Karubian J, Webster MS. 2023. Photoperiod and rainfall are associated with seasonal shifts in social structure in a songbird. Behav Ecol. 34(1):136–149.

[CIT0102] Wey T , Blumstein DT, Shen W, Jordán F. 2008. Social network analysis of animal behaviour: a promising tool for the study of sociality. Anim Behav. 75(2):333–344. doi:10.1016/j.anbehav.2007.06.020

[CIT0103] Wey TW , Blumstein DT. 2010. Social cohesion in yellow-bellied marmots is established through age and kin structuring. Anim Behav. 79(6):1343–1352. doi:10.1016/j.anbehav.2010.03.008

[CIT0104] Wild S , Alarcón-Nieto G, Aplin L. 2024. Social network data in wild great tits during ontogeny. Behav Ecol. doi:10.5061/dryad.x95x69ps8 PMC10941318 38495730

[CIT0105] Woodman JP , Cole EF, Firth JA, Perrins CM, Sheldon BC. 2023. Disentangling the causes of age-assortative mating in bird populations with contrasting life-history strategies. J Anim Ecol. 92(5):979–990. doi:10.1111/1365-2656.13851 36423201

