# Supplementary material for: The ontogeny of social networks in wild great tits (Parus major)
Source: Behav Ecol. 2024 Feb 29;35(2):arae011. doi: 10.1093/beheco/arae011 (PMC10941318; doi:10.1093/beheco/arae011)
Supplement: arae011_suppl_Supplementary_Material [file arae011_suppl_supplementary_material.docx]

Supplementary Information:

The ontogeny of social networks in great tit (*Parus major*) fledglings

Wild, Sonja^1, 2, 3†^; Alarcón-Nieto, Gustavo^1, 4, 5, 6^; Aplin, Lucy^1, 7, 8^

^1^ Cognitive and Cultural Ecology Research Group, Max Planck Institute of Animal Behavior, Am Obstberg 1, 78315 Radolfzell, Germany

^2^ Centre for the Advanced Study of Collective Behaviour, University of Konstanz, Universitätsstrasse 10, 78464 Konstanz, Germany

^3^ Department of Environmental Science & Policy, University of California Davis, One Shields Ave, Davis, CA-95616, United States

^4^ Department of Migration, Max Planck Institute of Animal Behavior, Am Obstberg 1, 78315 Radolfzell, Germany

^5^ International Max Planck Research School for Quantitative Behaviour, Ecology and Evolution, Am Obstberg 1, 78315 Radolfzell, Germany

^6^ Department of Biology, University of Konstanz, Universitätsstrasse 10, 78464 Konstanz, Germany

^7^ Department of Evolutionary Biology and Environmental Studies, University of Zurich, Winterthurerstrasse 190, 8057 Zurich, Switzerland

^8^ Division of Ecology and Evolution, Research School of Biology, Australian National University, 46 Sullivan’s Creek Road, Canberra, ACT 2600, Australia

^†^ Corresponding author: [swild@ucdavis.edu](mailto:swild@ucdavis.edu)

**Table S1: Data collection across seasons**

| **Period** | **Dates** | **Num. 48-hour periods** |
| --- | --- | --- |
| Summer 2020 | 05.05.-06.08.2020 | 14 |
| Autumn 2020 | 29.09.-15.10.2020 | 3 |
| Winter 2020 | 24.11.-08.12.2020 | 3 |
| Spring 2021 | 23.02.-11.03.2021 | 3 |

**On the interpretability of odds ratios:**

Odds ratios (OR) are a commonly reported outcome variable in statistical models that allow for the comparison of two proportions (e.g. Rita & Komonen, 2008). The OR refers to the change in odds of an event occurring per one-unit increase in the predictor variables, while all other predictors are held constant. If the odds ratio is 1, this implies that the predictor has no effect on the outcome variable. If the OR is greater than 1, it suggests an increase in the odds, and if below 1, it suggests a decrease in the odds. Applied to our results reported in analysis 5 (assessing network stability across seasons): The OR of summer associations predicting those in autumn are 12.38. This means that two birds who in summer spent all of their time together (association strength of 1) are 12.38 times more likely to spend all their time together in autumn, compared to those who were never associated in summer (association strength of 0), while all other covariates are held constant.

**Table S2: Variance inflation factors for predictor variables:**

|  | Predictor | GVIF | DF |
| --- | --- | --- | --- |
| Analysis 1 (Model 1) | Relationship type | 1.08 | 3 |
|  | Time since fledging | 1.02 | 1 |
|  | Space use overlap | 1.08 | 1 |
| Analysis 2:  (Model 2) | Age difference | 1.00 | - |
|  | Space use overlap | 1.00 | - |
|  | Weight difference | 1.00 | - |
| Analysis 3: (Model 3a) | Summed association with parents | 1.29 | - |
|  | Space use overlap | 1.29 | - |
|  | Time since fledging | 1.00 | - |
| Analysis 3: (Model 3b) | Summed association among parents | 1.07 | - |
|  | Space use overlap | 1.05 | - |
|  | Time since fledging | 1.02 | - |

**Table S3: Model outputs (odds ratio) for predictors for dyadic associations during transition to independence (Model 1)**

|  | Estimate | l-95% CI | u-95% CI | Rhat |
| --- | --- | --- | --- | --- |
| Intercept | 0.07 | 0.06 | 0.08 | 1 |
| R*: parent-offspring | 1.05 | 0.93 | 1.18 | 1 |
| R: peers | **1.11** | **0.99** | **1.23** | 1 |
| R: siblings | **1.23** | **1.08** | **1.39** | 1 |
| A: time since fledging | **1.15** | **1.12** | **1.18** | 1 |
| S: space overlap | **2.22** | **2.09** | **2.37** | 1 |
| R:A: age*parent-offspring | **0.97** | **0.88** | **1.08** | 1 |
| R:A: age*peers | **0.92** | **0.89** | **0.95** | 1 |
| R:A: age*siblings | **0.81** | **0.76** | **0.87** | 1 |

* R = relationship; A = age (time since fledging in days); S = space use overlap (complete to no overlap). Effect sizes for R are estimated relative to the baseline level (associations with non-parent adults).

**Table S4: Model outputs (odds ratio) for predictors for dyadic associations among non-sibling fledglings during transition to independence (Model 2)**

|  | **Estimate** | **Est. Error** | **l-95% CI** | **u-95% CI** | **Rhat** |
| --- | --- | --- | --- | --- | --- |
| Intercept | 0.08 | 1.09 | 0.07 | 0.09 | 1 |
| Space overlap | **2.28** | **1.05** | **2.09** | **2.49** | 1 |
| Age difference | 1.01 | 1.02 | 0.98 | 1.04 | 1 |
| Weight difference | 1.01 | 1.01 | 0.99 | 1.04 | 1 |
| Age difference:time since fledging | **0.95** | **1.01** | **0.93** | **0.98** | 1 |
| Weight difference:time since fledging | 0.99 | 1.01 | 0.97 | 1.01 | 1 |

**Table S5: Model outputs (odds ratio) for inheritance of parental social networks (Models 3a and 3b)**

|  |  | **Estimate** | **Est. Error** | **l-95% CI** | **u-95% CI** | **Rhat** |
| --- | --- | --- | --- | --- | --- | --- |
| Model 3a: associations between juvenile and non-parent adults | Intercept | 0.08 | 1.06 | 0.07 | 0.08 | 1 |
|  | **Space overlap** | **2.00** | **1.05** | **1.83** | **2.19** | 1 |
|  | Summed association with parents | 0.97 | 1.23 | 0.65 | 1.46 | 1 |
|  | **Summed association with parents:time since fledging** | **2.29** | **1.10** | **1.89** | **2.76** | **1** |
|  |  |  |  |  |  |  |
| Model 3b: associations among juveniles | Intercept | 0.08 | 1.09 | 0.07 | 0.09 | 1 |
|  | Space overlap | **2.21** | **1.05** | **2.02** | **2.41** | 1 |
|  | Summed association among parents | 1.13 | 1.11 | 0.91 | 1.39 | 1 |
|  | **Summed association among parents:time since fledging** | **1.24** | **1.06** | **1.10** | **1.39** | **1** |


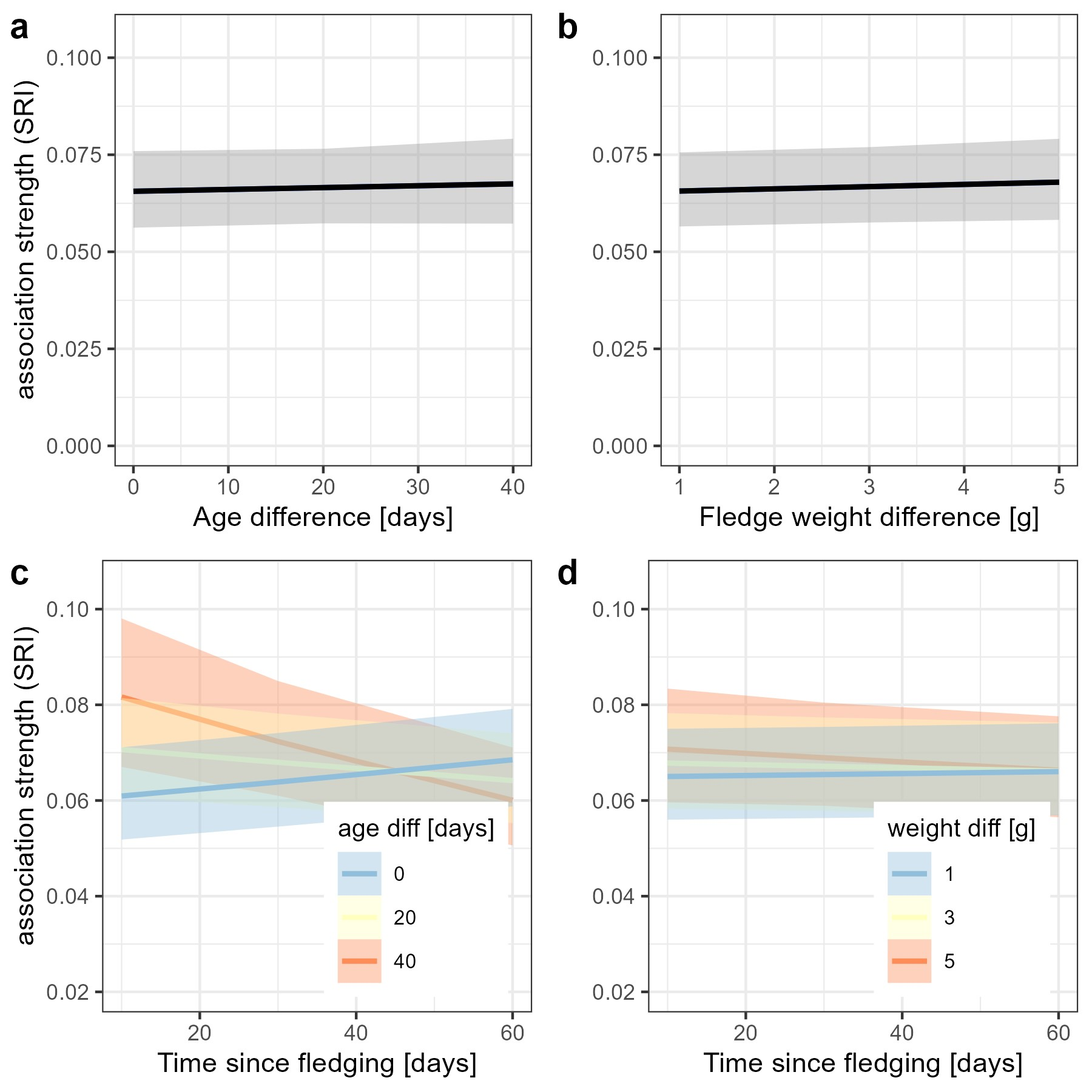


**Figure S1: Factors predicting association among non-sibling fledglings: a: Age similarity:** After controlling for space use overlap, there was no evidence that juveniles preferentially associated with those closer in age. **b: Weight similarity:** There was no evidence for preferred associations among juveniles similar in weight. **c: Age similarity over time:** With increasing age, juveniles’ association with those of similar age showed a marginal increase compared to those with larger age differences. **d:** **Weight similarity over time:** There was no evidence for preferred associations among those similar in fledge weight regardless of age of the focal individual.

**Table S6: Model outputs (odds ratio) for associations during the previous season predicting dyadic association strengths while controlling for space use and age (Models 5a-c)**

|  | **Season** | **Model** | **Estimate** | **Est. Error** | **l-95% CI** | **u-95% CI** | **Rhat** |
| --- | --- | --- | --- | --- | --- | --- | --- |
| Intercept | autumn | 5a | 0.06 | 1.23 | 0.04 | 0.09 | 1 |
|  | winter | 5b | 0.03 | 1.22 | 0.02 | 0.05 | 1 |
|  | spring | 5c | 0.03 | 1.27 | 0.02 | 0.05 | 1 |
| Association prev. season | autumn | 5a | **12.38** | **2.17** | **2.67** | **54.65** | 1 |
|  | winter | 5b | **3.07** | **1.32** | **1.78** | **5.34** | 1 |
|  | spring | 5c | **2.26** | **1.29** | **1.37** | **3.76** | 1 |
| Focal age* | autumn | 5a | 1.18 | 1.26 | 0.74 | 1.82 | 1 |
|  | winter | 5b | 0.92 | 1.27 | 0.58 | 1.49 | 1 |
|  | spring | 5c | 1.26 | 1.35 | 0.69 | 2.30 | 1 |
| Space overlap | autumn | 5a | **2.70** | **1.21** | **1.88** | **3.92** | 1 |
|  | winter | 5b | **14.05** | **1.10** | **11.70** | **16.90** | 1 |
|  | spring | 5c | **8.67** | **1.10** | **7.23** | **10.35** | 1 |
| Association prev. season: age* | autumn | 5a | **0.06** | **3.42** | **0.01** | **0.64** | 1 |
|  | winter | 5b | 0.88 | 1.52 | 0.38 | 2.00 | 1 |
|  | spring | 5c | **2.15** | **1.48** | **0.99** | **4.65** | 1 |

* first-year versus adult

# References

Rita, H., & Komonen, A. (2008). Odds ratio: An ecologically sound tool to compare proportions. In *Annales Zoologici Fennici* (Vol. 45, Issue 1, pp. 66–72). Finnish Zoological and Botanical Publishing Board. https://doi.org/10.5735/086.045.0106
